# Rare Cases in Obstetrics

**Published:** 1894-07

**Authors:** William F. Holt

**Affiliations:** Macon, Ga.


### RARE CASES IN OBSTETRICS.*

By WILLIAM F. HOLT, M.D., Macon, Ga.

The following cases are to my mind so unusual in the practice of the general practitioner that it occurred to me to present them at this meeting for comment and discussion. I shall give a brief and concise history of each case, with the treatment adopted.

Case 1.—Mrs. A. B., Jewess, mother of six children, good family history, offspring all healthy, previous labors normal in every respect; consulted me January 11, 1892, stating that she quickened at the usual time, about the expiration of the fourth month; that the breast enlarged, and that there was a gradual increase in size, but that she had not felt the motion of the fetus for several days. Upon examination I could map out the uterus, and expressed the opinion that the fetus was blighted; that the placenta was retained probably vitalized; that it would sooner or later be thrown off. She was incredulous, but accepted my diagnosis. Her health continued good; was able to discharge her household duties as usual. Heard nothing further from her until I was summoned to see her, March 23d, at 11 P. M. She then informed me that she had had pains since early morning, recurring regularly and attended with slight sanguineous discharge; that they had increased in severity and occurred now every five minutes. Upon examination, found the os fully dilated, and in a short time the fetus was expelled dry and shrivelled, corresponding to what we would expect at four months. Finding it impossible to express or remove the placenta, and the hemorrhage being considerable, I tamponed through a cylindrical speculum with carbolized cotton, ordered thirty minims of fluid extract ergot given every three hours, and morphine if unable to sleep. Upon calling to see her the following morning, I found that the pains had continued through the night, that she had taken the opiate, causing her to sleep fairly well, but had no further hemorrhage. Upon removing the tampon, I found the placenta (four months) detached and in vagina. Gave her a douche of carbolic acid and water, ʒ i to O i, and placed her in

*Read before Medical Association of Georgia, April, 1894.

bed; repeated the douche for three consecutive mornings; she had no increase of temperature; slight lochial discharge; her recovery rapid and complete.

CASE 2.—Mrs. B. C., age thirty-one, neurotic temperament; mother of two children, the youngest eight years; has not menstruated since the birth of last child; had a leucorrheal discharge in August last. Saw her in December, when she told me she was pregnant. Upon examination, I could hear distinctly the fetal heart and feel the impulse of child through the abdominal walls. She was delivered of a female infant February 28, 1894, small but perfect in form; nothing abnormal in her labor; lying-in was attended with no unpleasant symptom; lochia continued for four weeks; no secretion of milk.

CASE 3.—Mrs. C. D., age thirty-one, good family history and has always enjoyed excellent health; has two bright and vigorous children, the youngest four and one-half years of age. Menstruated regularly up to March 27, 1893, continued the usual time, five days; no further menstruation until July 23, 1893, when it was very profuse. Treated her for threatened miscarriage: opiates, viburn. prunifolii, rest in bed, etc. Was confined to her room two weeks; she was then able to resume her household duties, reported at my office at the expiration of a month, and occasionally afterwards, stat. ing that she had not quickened and that there was no increase in her size, breast full and very tender. I gave her the opinion that the ovum was blighted and the placenta would sooner or later be thrown off. She had a slight leucorrheal discharge in January, 1894. On the 28th of March she was seized with pains at 4 A. M., with slight hemorrhage, the pains increasing in severity. I was summoned at 9 A. M., found the os fully dilated and the placental mass distinctly felt. As the pains were then of sufficient force, I thought to expel the placenta; I awaited further result; at 12 M. the pains ceased, when I gave her ʒ i fluid extract ergot, which had the effect of increasing them, and at 3:30 P. M. the placental mass was expelled entire, twelve months and one day from the date of last menstruation, the membranes very thick and tough. Gave injection bichloride solution 1 to 2000 for three consecutive mornings. Nothing abnormal occurred, and she rapidly convalesced.

Case 4.—Mrs. H. G., aged thirty-six, has always enjoyed excellent health; four children, all strong and healthy; her previous labors unattended with difficulty, normal in every respect. Consulted me in latter part of November, 1892; had missed catamenia for two months, felt unusually languid, was compelled to remain in bed, as when she attempted to sit up she felt "dizzy and faint"; had frequent and annoying pains in the lumbar region. Made an instrumental examination. Uterus in normal position; nothing revealed which could account for the symptoms which she had detailed. She was confined to her room most of the time, occasionally sitting up a few hours. On January 1, 1893, at 3 P. M., I was hastily summoned; was informed that she raised up in bed suddenly and lifted her youngest child, whom she thought was in convulsions, after which she was having labor pains at regular intervals, accompanied with slight hemorrhage, which continued increasing in severity until the fetus was expelled, completely enveloped in the membranes. Upon opening the sac and examining the fetus I concluded it was eight weeks. I congratulated myself upon the happy termination of it. Patient was bright and cheerful. I remained in the room, as is my invariable custom, for one hour, and directed the nurse to thoroughly cleanse the external genitals with a warm solution of bichloride 1 to 2000. After seeing my patient placed comfortably in bed, I was preparing to leave the house, when she informed me that she was very faint. Upon going to the bed I found she was bleeding profusely, lowered her head, introduced ice and kneaded the womb, gave her ʒi Squibbs's fluid extract of ergot; the hemorrhage continuing, and being unable to express or remove the placenta, and her condition so feeble that I thought further efforts would be hazardous, I introduced a cylindrical speculum and tamponed her with carbolized cotton; her condition at this time was so critical that Dr. C. H. Hall was summoned to see her. Upon his arrival she had rallied, and after consultation we decided to continue the ergot through the night 30 minims every three hours, and morphine if restless. The following morning I removed the tampon, but was unable to remove the placenta either with forceps or my fingers; gave her a sublimate solution 1 to 2000, and reapplied the tampon. For two consecutive mornings the same process was repeated with like result.

After further consultation we determined that it would be hazardous in her debilitated condition to curette the womb, but decided to keep her in bed and give her iron and a liberal diet and build up her system. After the lapse of six weeks she had improved sufficiently to be placed under chloroform, when Dr. W. B. Gilmer curetted the womb, assisted by Dr. Hall and myself, removing a large piece of placenta which was vitalized and attached to the fundus.

He then packed the womb with iodoform gauze, allowed it to remain four days, removing a small portion each day. She had no further hemorrhage and continued to improve. After the expulsion of the fetus completely enveloped in the membranes, I naturally concluded that nothing further remained, and had I left my patient she would probably have lost her life.

